# Novel Divinyl-Flanked Diketopyrrolopyrrole Polymer, Based on a Dimerization Strategy for High-Performance Organic Field-Effect Transistors

**DOI:** 10.3390/polym15234546

**Published:** 2023-11-27

**Authors:** Jinyang Chen, Jie Zhou, Na Li, Yubing Ding, Shiwei Ren, Minfeng Zeng

**Affiliations:** 1 Zhejiang Key Laboratory of Alternative Technologies for Fine Chemicals Process, Shaoxing University, Shaoxing 312000, China; 2023000069@usx.edu.cn (J.C.);; 2 Zhuhai-Fudan Innovation Institute, Hengqin 519000, China

**Keywords:** polymer semiconductor, polymerization, high mobility, organic transistor, organic electronics

## Abstract

In this communication, we report a novel acceptor structural unit, TVDPP, that can be distinguished from classical materials based on TDPP structures. By designing a synthetic route via retrosynthetic analysis, we successfully prepared this monomer and further prepared polymer P2TVDPP with high yield using a Stille-coupling polymerization reaction. The polymer showed several expected properties, such as high molecular weight, thermal stability, full planarity, small π−π stacking distance, smooth interface, and so on. The absorption spectra and energy levels of the polymer were characterized via photochemical and electrochemical analysis. The organic field-effect transistor (OFET), which is based on P2TVDPP, exhibited excellent carrier mobility and an on/off current ratio of 0.41 cm^2^ V^−1^ s^−1^ and ~10^7^, respectively, which is an important step in expanding the significance of DPP-based materials in the field of optoelectronic devices and organic electronics.

## 1. Introduction

**Figure 1:**
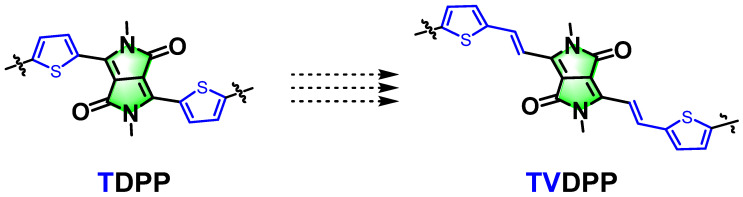
A strategy for designing a novel acceptor structural unit by inserting double bonds into the most widely studied classical molecular structure of TDPP.

Organic semiconductor (OSC) materials, especially organic polymer semiconductors, have attracted great attention as carrier transport materials [1,2]. Research in this area was awarded the Nobel Prize in Chemistry in 2000, and, over the past 20 years, a variety of polymer systems have been developed and applied to many applications, including organic solar cells, organic thermoelectrics, organic luminescence, sensors, organic electrochemical transistors, and so on [3,4,5,6,7,8]. A fundamental component application in the field of organic electronics is the organic field-effect transistor (OFET), which can support a variety of functionalities to fulfill the requirements of various applications [9,10]. Among them, the most widely investigated core material system for controlling the performance of devices is the diketopyrrolopyrrole-thiophene (TDPP)-based system, which can be conveniently used as an acceptor building block for polymerization reactions with electron donors or electron donors, to prepare donor-acceptor-type polymers or donor–donor-type polymers [11,12,13,14]. Many excellent polymer structures have been reported and show high electron mobility and/or hole mobility [15,16,17]. However, the scarcity of new acceptor building unit structures limits the continued development of this field. Among TDPPs, thiophene tends to act as an electron-donating unit that is attached to the DPP moiety via a single bond [18]. Although the replacement of the thiophene unit with an aromatic ring, such as a pyridine ring, benzene ring, etc., has been reported, direct modification of the linked single bond has not been reported. We envisage breaking this linkage and introducing olefins by means of retrosynthetic analysis to prepare new monomeric building blocks (Figure 1). The introduction of olefins does not theoretically reduce the planarity of the material and can effectively expand the conjugation length within the molecular structure, which is expected to lead to the preparation of new potential acceptor building blocks to enrich the structural variety of the molecular library.

## 2. Materials and Methods

Materials: The raw materials such as fumaroyl chloride, triphenylphosphine, toluene-4-sulphonic, etc., and solvents such as methanol, tetrahydrofuran (THF), and hexane were obtained from Merck (Rahway, NJ, USA). Hexamethylditin and 3,3,6,6-tetramethyl-9-(1,2,3,4-tetrahydroxybutyl)-4,5,7,9-tetrahydro-2H-xanthene-1,8-dione (Pd_2_(dba)_3_) were purchased from Suzhou SunaTech (Suzhou, China). 

Synthesis of Compound **2**: 2-Octyldodecylamine (7.44 g, 25 mmol) was added to a three-necked flask (250 mL) with nitrogen exchange, followed by the addition of triethylamine (2.02 g, 20 mmol). Then, 100 mL of dry THF was added to the flask and the mixture was stirred thoroughly for 0.5 h under ice-bath conditions. After the reaction system had cooled completely, compound 1 (1.53 g, 10 mmol) in THF (10 mL) was added dropwise to the reaction flask, and the dropwise process was continued for 40 min. The mixture continued to be stirred at room temperature for 2 h, after which stirring was terminated. The mixture was then diluted with water (50 mL) and extracted with ethyl acetate (3 × 80 mL). The organic phases were combined and washed three times with saturated brine (3 × 100 mL). The organic phases were separated via column chromatography after drying, filtration, and concentration (ethyl acetate:petroleum ether = 1:3) to produce 5.06 g of a white solid product at a 75% yield. 

Synthesis of compound **3**: Intermediate compound **2** (4.45 g, 6.6 mmol) and isopropenyl acetate (50 mL) were added to a round-bottomed flask (150 mL). Subsequently, p-toluenesulfonic acid (7.0 mL) was added and the mixture was warmed up to 95 °C. The reaction was carried out via condensation reflux for 5 h. The formation of the major products was monitored via thin-layer chromatography (TLC). After the reaction was brought down to room temperature, the solvent in the flask was spun off by distillation under reduced pressure, followed by washing with 5% HCl solution in water (3 × 100 mL) and saturated sodium bicarbonate solution three times (3 × 100 mL), sequentially. The organic phase was dried, filtered, and concentrated under reduced pressure. The product was collected via column chromatography (ethyl acetate:petroleum ether = 1:5) to give 3.35 g of brown liquid at a 67% yield.

Synthesis of compound **4**: Compound **3** (1.52 g, 2.0 mmol), triphenylphosphine (629.5 mg, 2.4 mmol), and pyridinium 4-methylbenzenesulphonate (251.3 mg, 1.0 mmol) were added to a pressure-resistant flask, followed by the addition of 40 mL of a solvent mixture of THF and acetonitrile (3:1). The flask was freeze-evacuated to maintain negative pressure inside the flask. The reaction system was gradually warmed up to 100 °C and then left overnight. Extraction was carried out using dichloromethane (3 × 50 mL), followed by drying using sodium sulfate. Filtration, concentration, and purification by column chromatography (ethyl acetate: petroleum ether = 1:5) produced 580 mg of yellow oily liquid at a 40% yield. 

Synthesis of compound TVDPP: Compound **4** (725.7 mg, 1.0 mmol), 5-bromothiophene-2-carboxaldehyde (477.6 mg, 2.5 mmol), L-proline (23.1 mg, 0.2 mmol), and triethylamine (4 mmol) were introduced into a flask (100 mL), followed by the addition of a mixture of toluene and methanol (5:1). Oxygen was removed by repeated freeze-pumping under nitrogen protection. The mixture was reacted at room temperature for 10 h and the reaction endpoint was monitored via TLC. The reaction system was extracted with dichloromethane (3 × 50 mL) and then dried using sodium sulfate. The organic phase was filtered, mixed with silica gel powder, and spun-dried, then it was purified via column chromatography (dichloromethane: petroleum ether = 2:1). The resulting crude product was then settled to give 460.3 mg of brown solid at a 43% yield. 

The corresponding nuclear magnetic resonance (NMR) spectra (^1^H NMR and ^13^C NMR) of all organic small molecules are shown in Figures S1–S8 in the Supplementary Materials, respectively. The results of the mass spectral characterization of the materials are shown below, in order: compound **2**, calculated for C_44_H_87_N_2_O_2_^+^: 675.67676. Found: 675.67671; compound **3**, calculated for C_48_H_91_N_2_O_4_^+^: 759.69788. Found: 759.69713; compound **4**, calculated for C_48_H_89_N_2_O_2_^+^: 725.69241. Found: 725.69172; compound TVDPP, calculated for C_58_H_90_Br_2_N_2_O_2_S_2_^+^: 1071.48683. Found: 1071.48743.

Synthesis of the polymer: Under nitrogen protection, TVDPP (100.0 mg, 0.093 mmol), hexamethyldistannane (30.5 mg, 0.093 mmol), Pd_2_(dba)_3_ (2.6 mg, 2.8 μmol), and tri(o-tolyl)-phosphine (P(o-tol)_3_, 6.8 mg, 22.3 μmol) were sequentially added to a pre-dried 10 mL Schlenk flask. Anhydrous tetrahydrofuran solvent (5 mL) was added to the mixture, which was subsequently deoxygenated via a refrigerated pumping cycle under nitrogen. Stirring was initiated and the reaction mixture was allowed to react at 120 °C for 24 h. After returning to room temperature, the mixture was transferred to a beaker containing methanol (100 mL) and stirred for 2 h at room temperature. Further filtration afforded solid particles, which were subsequently loaded into a Soxhlet extractor. Sequentially, methanol, acetone, ethyl acetate, and hexane were utilized for 10 h to remove the oligomers. The extract was subsequently run with chloroform and then concentrated to remove the solvent. Precipitation was again carried out via methanol (150 mL) for 3 h. The solid was filtered and subsequently dried under vacuum (120 °C) to produce a black powder (P2TVDPP, 91% yield).

Organic field-effect transistor (OFET): The device was manufactured on the 0.6 cm × 0.6 cm SiO_2_/Si substrate, with a bottom-gate bottom-contact architecture. After ultrasonic cleaning in deionized water, ethanol, and isopropanol for 10 min each, the substrate was blown dry with nitrogen, then the source and drain electrodes were deposited via thermal evaporation. The channel width/length of the OFET device was 1400/30 µm. The polymer P2TVDPP was pre-dissolved in chlorobenzene at an elevated temperature (60 °C) and stirred overnight to allow for the homogeneous dispersion of the solution. The polymer film was deposited onto the substrate at a fixed spin-coating rate of 3000 rpm under ambient conditions. The polymer films were then annealed at 150 °C for 30 min to remove the chlorobenzene solvent.

Characterization: The molecular weight and degree of dispersion of the P2TVDPP were evaluated via gel permeation chromatography at 150 °C (eluent: trichlorobenzene, Shimpack GPC-80M, Kyoto, Japan). Thermal stability measurements were conducted under nitrogen (TGA2, Mettler, Switzerland). Elemental analysis was achieved using the CHN mode of an organic elemental analyzer (FlashSmart, Thermo Scientific, Waltham, MA, USA). Photochemical characterization was performed on a UV-visible spectrometer (Cary5000, Agilent, Santa Clara, CA, USA). The concentration of the solution in chloroform solvent was approximately 0.5 mg/mL. The polymer semiconductor solution was spin-coated onto pre-cleaned quartz plates (1.0 cm × 3.0 cm) and then the solvent was allowed to evaporate slowly to prepare the film. Electrochemical tests were carried out in acetonitrile solution containing tetrabutylammonium hexafluorophosphate (0.1 M), at a scanning rate of 100 mV/s. An Ag/AgCl electrode, glassy carbon electrode, and platinum electrode were used as a reference electrode, a working electrode, and a counter electrode, respectively. A drop of 5 µL of polymer chloroform solution was pipetted onto a glassy carbon electrode with a hole of 4 mm in diameter and allowed to evaporate slowly. The HOMO energy level was extracted from the onset of the oxidative peak by estimating the HOMO energy level according to the equation: E_HOMO_ = −4.80 − (E_ox_^onset^ − 0.40) eV, where E_ox_^onset^ was relative to the measured oxidation potential value of the Fc/Fc^+^ redox pair. The LUMO energy levels were calculated from the onset of the reductive peak using the equation: E_LUMO_ = −4.80 − (E_red_^onset^ − 0.40) eV. OFET characterizations were carried out in a glove box using the 4200-semiconductor system (Keithley, Cleveland, OH, USA). The hole mobility (*µ*) is calculated as: *µ* = ($\frac{\partial \surd {|I}_{DS}|}{\partial V_{GS}}$)^2^ ∙ $\frac{2L}{WCi}$, where W and L are the width and length of the device channel, respectively; I_DS_ and V_GS_ are the source-drain current and gate voltage, respectively; Ci is the capacitance per unit area of the gate dielectric layer. Atomic force microscopy (AFM) measurement of the sample in the thin film annealed state was carried out using a Nanoscope V instrument (Burker, Germany). The sample was tested in an identical manner to that employed for OTFT device performance analysis. The 2D-grazing-incidence wide-angle X-ray scattering result was obtained from the 14B/15U beamline station (Synchrotron Radiation Facility, Shanghai, China).

## 3. Results

### 3.1. Synthesis Routes to Producing P2TVDPP Polymer

**Scheme 1:**
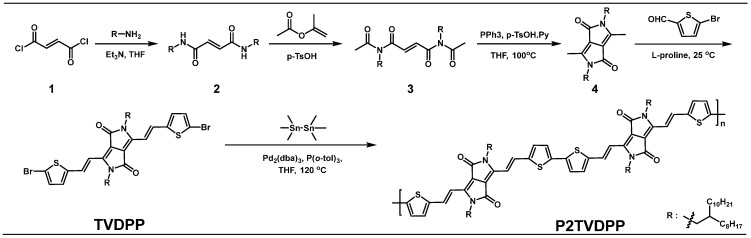
Synthesis process and experimental conditions based on a four-step reaction for the preparation of the monomer TVDPP and subsequent preparation of the polymer P2TVDPP.

The synthetic route and experimental conditions from monomer to polymer are presented in Scheme 1. The TVDPP molecule features a symmetric structure with two five-membered rings that are fused together. The olefin itself exhibits a trans configuration that restricts the conformation of the molecule while the overall structure shows well-defined planarity and rigidity, which facilitates efficient carrier transport. The molecule contains two strong electron-withdrawing groups, forming carbonyl (C=O), which makes it a good acceptor unit [19]. The material possesses better planarity than the commonly found TDPP-based acceptor units. The crystal results of the TDPP material show that it could reveal homogeneous polycrystalline phenomena, depending on the type of alkyl chains and environmental conditions. The thiophene, which is connected to the DPP backbone by a single bond on both sides, can show a variety of isomers by the presence of various torsional phases, such as the homo and hetero sides [20,21]. As a result, subsequent polymers based on TDPP can exist in different connection modes, including head-to-head, head-to-tail, and tail-to-tail connections during the polymerization process. To some extent, this reduces the purity of the material and limits the planarity and regularity of the material, thus decreasing the carrier mobility it can assume. In this study, the introduction of olefins was relied upon to form intramolecular conformational locks, which strongly circumvents the problem of isomers. It is worth noting that the introduction of olefins is also advantageous for extending the conjugation lengths contained in the materials, which facilitates further control of the frontier orbital energy levels of the materials. The position of the N of the side chain facilitates the introduction of long alkyl chains, which improves the solubility and processability of the material. The palladium-catalyzed self-polymerization of the monomer TVDPP led to the preparation of the polymeric material P2TVDPP. The acceptor dimerization strategy is widely used in classical acceptor building blocks to form a variety of polymers such as TDPP-TDPP, which is conducive to the study of the intrinsic carrier mobility properties of such materials and the enrichment of the family of such materials [22]. The polymerization process of TDPP-based materials that has been reported in some of the literature requires the use of chlorobenzene as the reaction solvent, which can be attributed to the inferior solubility of the material. In our case, we successfully prepared an extremely high molecular weight material using tetrahydrofuran (THF) as a solvent for the polymerization reaction. THF is a green, low-cost solvent with significantly reduced environmental and human hazards compared to toxic reagents containing chlorine [23].

**Figure 2:**
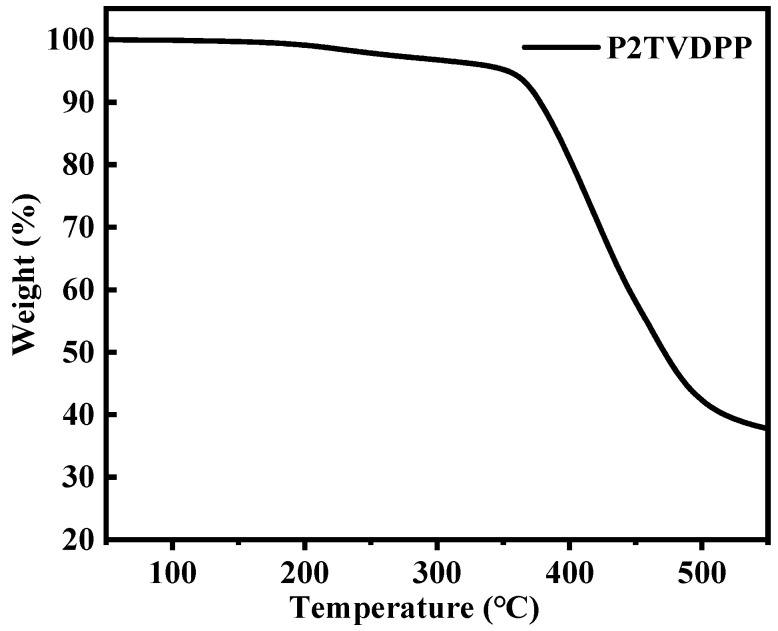
Analysis of the thermal weight loss of the polymer P2TVDPP with temperature rise.

The elemental and molecular weight analyses of the polymers that were formed with alternating arrangements of donor-acceptor architectures are shown in Table 1. The contents of the three elements C, N, and H are all within one percent of the smallest repeating unit (i.e., 2TVDPP). This indicates that we successfully prepared the expected products with very high purity, a process that relies on the Soxhlet extraction method to effectively remove impurities such as catalysts and ligands from the mixture system. The number average molecular weight of the polymers was assessed to be 35.58 kDa using high-temperature GPC, indicating that each polymer chain contains an average of approximately 33 TVDPP units. The ratio based on the weight average molecular weight to Mn showed a narrow molecular weight distribution for the molecule, with a polydispersity index (PDI) of only 1.89. Distinguishing this from the majority of TDPP-based polymers, which tend to feature PDIs in the range of 2–4, this is indicative of the homogeneity of the individual chains contained in our material. Characterization data, such as the Z-mean molecular weight (Mz) and the molecular weight of the highest peak (Mp) of the polymer, are shown in Figure S9. The macromolecule was further analyzed for thermal decomposition and the results are shown in Figure 2. The polymer lost 5% and 10% of its mass at 355 °C and 380 °C, respectively, indicating that it possesses good thermal stability properties. 

### 3.2. Density Functional Theory (DFT) Calculation of P2TVDPP

We employed DFT simulations to investigate the theoretical conformation of the material with the test set under the B3LYP/6-31G(d) moiety. Calculations of the energy levels and planarity of the molecular orbitals of the material front were carried out via geometry optimization [24,25]. Since the non-conjugated components of the side chains hardly affect the overall planarity and energy levels of the material, methyl substitution is utilized here in place of the long aliphatic chains. To further reduce the computational cost and time, polymers with more than 30 repeating units were simplified to dimers with only two repeating units, an approach that has been accepted elsewhere in much of the literature [26,27]. 

**Figure 3:**
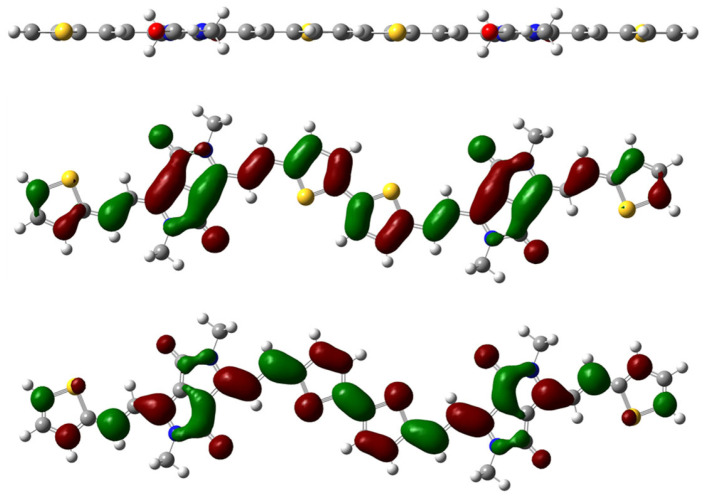
Top view of the optimized conformation of the methyl-substituted dimer of P2TVDPP (**top**); (**middle**) side view and the HOMO map of the dimer; (**bottom**) the LUMO map of the dimer.

In terms of the designed and envisaged properties, the most stable configuration of the dimer possesses excellent planarity. The olefin adopts a trans configuration, while the thiophene is connected to the thiophene by a head-to-tail attachment pattern. As shown in the top view of the molecular structure in Figure 3, the torsion angle of either the thiophene–thiophene ring or the five-membered ring–olefin linkage is extremely small, with an overall angle that is smaller than 1°. The good planar regularity of the polymer system effectively enhances the π–π stacking, which is conducive to the enhancement of the intra- and intermolecular charge transport of carriers, leading to the formation of potential materials with high mobility [28]. The highest occupied molecular orbital (HOMO) and lowest unoccupied molecular orbital (LUMO) energy levels of the polymer are fully delocalized on both the bis-DPP acceptor molecule and the π-conjugated bridge group and thiophene donor (Figure 3). According to our theoretical calculations, the HOMO and LUMO energy levels of the dimer are −4.67 eV and −3.06 eV, respectively, corresponding to an energy band gap of 1.61 eV. Considering that its energy levels are more compatible with the gold electrode, its transport ability regarding hole carriers was expected to be satisfactory.

### 3.3. Photochemical and Electrochemical Properties of P2TVDPP

**Figure 4:**
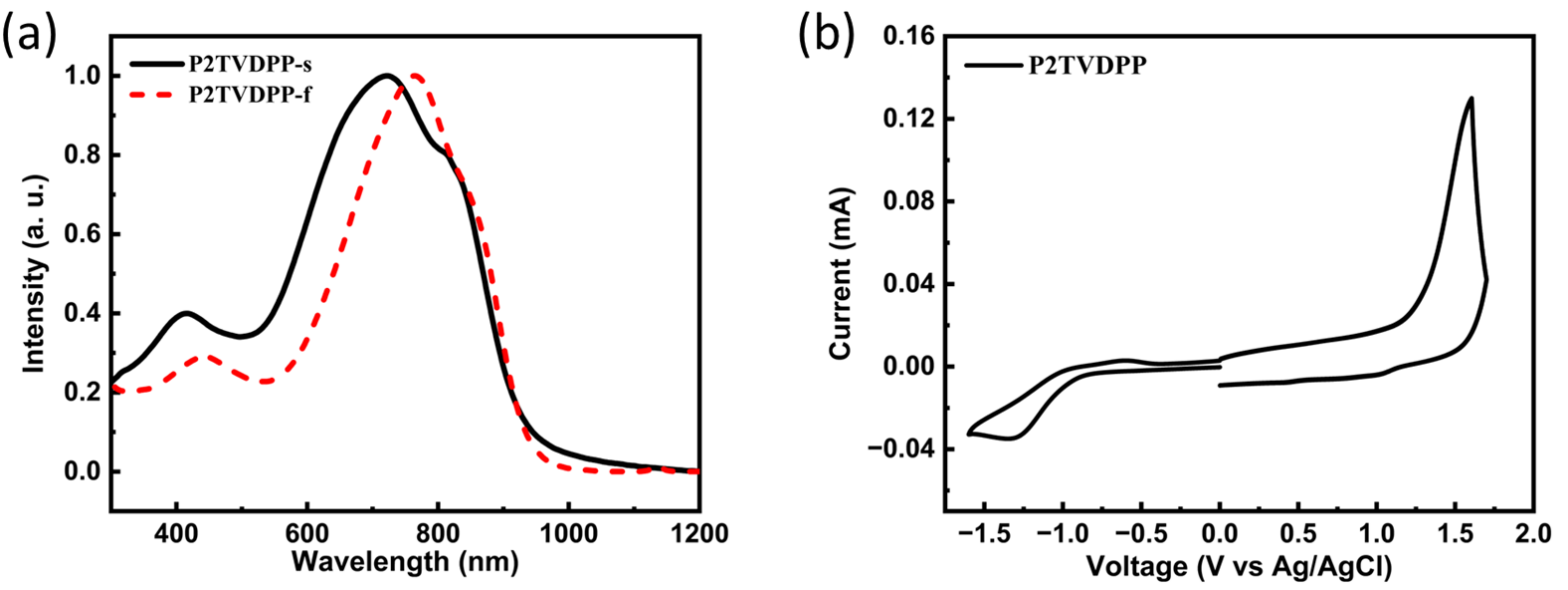
(**a**) UV-vis absorption spectra of polymer P2TVDPP in solution (black) and film (red); (**b**) cyclic voltammetry spectra of the polymer P2TVDPP film.

The UV-Vis absorption spectrum of P2TVDPP in chloroform exhibits an intense absorption band, with a maximum in the near-IR region at 1000 nm (Figure 4a). The absorption spectra in both states show that the onset of absorption is at ~1000 nm, suggesting the presence of long-range ordering as well as intermolecular interactions in both a solution and a solid state. Based on the position of the onset absorption peak in the solid state, the energy band can be deduced to be only 1.26 eV, and the narrow band gap can significantly improve the intramolecular charge transfer of the material, thereby increasing the mobility of charge carriers. The maximum absorption peaks in the solution and film phases are 723 nm and 768 nm, respectively, and the 45 nm of bathochromic shift is associated with stronger stacking in the solid state. To further investigate the redox properties and frontier molecular orbital energies of the materials, the polymers in the thin film state were then measured using cyclic voltammetry (Figure 4b). The polymers showed more pronounced oxidation properties, which may lead to their potential use as materials for hole transport compared to electron transport. The HOMO and LUMO energy levels of the material can be calculated from the internal standard ferrocene/ferrocene (Fc/Fc^+^) redox pairs and the positions of the onset oxidation and reduction peaks. The HOMO and LUMO energy levels of the material are −5.52 and −3.52 eV, respectively, which corresponds to an energy level difference of 2.00 eV. The difference between the bandgap calculated from the electrochemical measurements and the optical bandgap is nearly 0.70 eV, which is much closer to the energy level difference obtained from theoretical simulations.

### 3.4. P2TVDPP-Based OFET Device Performance 

**Figure 5:**
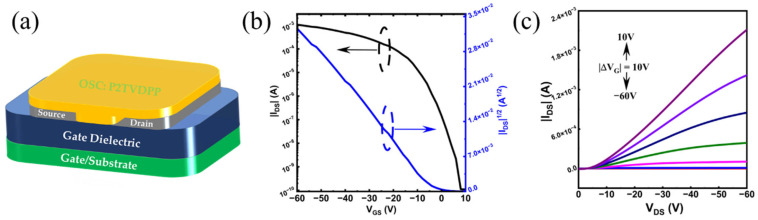
(**a**) Device configuration; (**b**) transfer and (**c**) output curve of a P2TVDPP-based device. The seven colored lines from bottom to top represent the different voltages.

We fabricated a bottom-gate–bottom-contact (BG-BC) configuration for the structured OFET devices based on P2TVDPP to characterize the charge transport behavior of the materials, and the corresponding device configuration is shown in Figure 5a. The saturation mobility (*µ*) is calculated as *µ* = ($\frac{\partial \surd {|I}_{DS}|}{\partial V_{GS}}$)^2^ ∙ $\frac{2L}{WCi}$. Table 2 summarizes the hole mobility extracted from the transfer characteristic curve (Figure 5b). The average hole mobility for a set of eleven devices is as high as 0.38 cm^2^ V^−1^ s^−1^, which is sufficiently good for a P-type material and meets the requirements for a wide range of applications such as electronic labels, sensing, and so on. The maximum hole mobility of the polymer occurs at 0.41 cm^2^ V^−1^ s^−1^, with optimized channel width and annealing temperatures. The devices also offer excellent performance with a low threshold voltage (V_th_) and sensitive switching ratios (I_on_/I_off_). It is worth noting that switching ratios close to the seventh power of 10 distinguish most TDPP-based polymer materials, which are typically within the fifth power of 10 [29]. The output profile, based on a P2TVDPP device, is illustrated in Figure 5c.

### 3.5. Morphological and Grazing-Incidence Wide-Angle X-ray Scattering Characterization of the P2TVDPP Film

**Figure 6:**
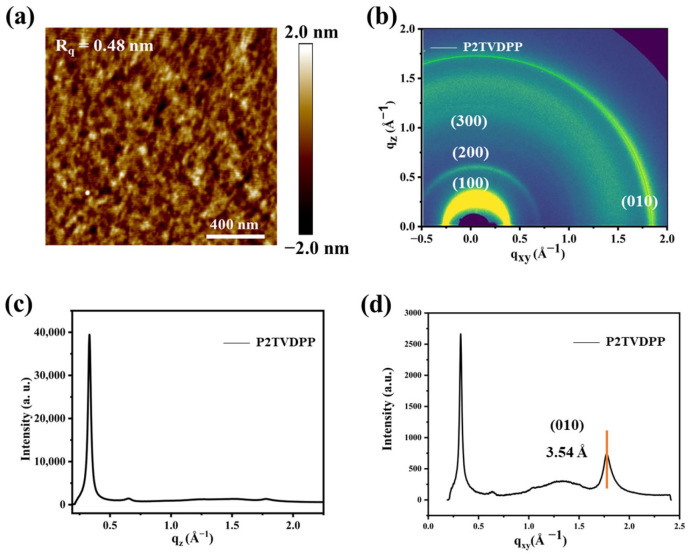
(**a**) AFM height images of polymer annealed films; (**b**) 2D-GIWAXS (top) and (**c**,**d**) 1D-GIWAXS profile of the annealed films of P2TVDPP.

Figure 6a illustrates the AFM height image of the annealed polymer film under optimal OFET device performance conditions. The root-mean-square roughness (RMS) of the polymer film P2TVDPP is only 0.48 nm, and the smooth interface reduces the defect density and facilitates efficient charge transfer. Furthermore, we investigated the relationship between the crystallinity of the annealed polymer film and the performance of OFET devices via 2D grazing-incidence wide-angle X-ray scattering (2D-GIWAXS). As illustrated in Figure 6b, the polymer presents third-order diffraction peaks, including (100), (200), and (300), and adopts the edge-on stacking mode. The d-d distance was calculated to be 19.96 Å by substituting Bragg’s equation, dsinθ = nλ, based on the angle corresponding to the diffraction peaks. The π–π stacking distance of P2TVDPP was estimated to be 3.54 Å, which was determined by basing the calculations on the out-of-plane (010) diffraction peaks (Figure 6c,d). The stacking distance is shown to be closer than that of most polymers, based on the TDPP structure [20], and the close π–π stacking facilitates excellent carrier transport capability.

## 4. Discussion

The polymers prepared in this work show good hole mobility, which is related to the coplanarity of the material itself and the regular alternating donor–acceptor arrangement. In order to maximize the planar regularity of the materials and to reduce the intramolecular torsion angles, much research has focused on the formation of intramolecular conformational locks through the introduction of weak interaction or conformational control groups. Kim et al. introduced an S...O weak interaction force and utilized space steric hindrance to significantly reduce the number of isomers and improve the overall planarity of the material, which exhibited a hole mobility as high as 0.18 cm^2^ V^−1^ s^−1^ [30]. Che et al. effectively improved the coplanarity of the material through non-covalent interactions, such as S...N and S...F, thereby preparing transistor materials with high carrier mobility [31]. In contrast to the weak interaction of non-covalent bonds, the introduction of functional groups such as alkenes or alkynes produces a more pronounced effect on the coplanarity and stacking of materials, resulting in the formation of films with a high degree of crystallinity [32,33,34]. The newly synthesized monomer TVDPP was also designed for the purpose of controlling thiophene orientation and overall material planarity [21]. In our next work, we will adopt the introduction of other strong acceptor units and strong electron-withdrawing atoms to prepare materials with both hole and electron mobility, to further enhance the potential of this material for organic optoelectronic applications.

## 5. Conclusions

In summary, this study reports a synthetic route to manufacturing monomer TVDPP with a new structure, which can be used as a potential acceptor group for polymerization to prepare organic semiconductor materials with long-conjugated architectures. Based on this monomer, we developed a complete green preparation route and avoided the use of chlorine-containing reagents. The polymer P2TVDPP exhibits good thermal stability and high solubility, which facilitates its material processing. Theoretical simulations show that the molecular arrangement of P2TVDPP is planar, which facilitates carrier transfer in the main chain. The results of our GIWAXS-based investigations indicate the existence of a close stacking pattern between the polymer chains, which further enhances the carrier transfer ability. Such macromolecular materials show hole transport rates of up to 0.41 cm^2^ V^−1^ s^−1^ in the OFET device.

## Figures and Tables

**Table 1:** The molecular weight of P2TVDPP and the ratio of elements it contains.

	Mn	Mw	PDI	C	H	N
	(kDa)	(kDa)		(%)	(%)	(%)
repeating unit ^1^	−	−	−	76.43	9.95	3.07
P2TVDPP	35.58	61.97	1.89	76.85	9.33	2.98

^1^ The molecular formula of the smallest repeating unit: C_58_H_90_N_2_O_2_S_2_.

**Table 2:** Hole transport behavior of the P2TVDPP-based OFET.

Organic Semiconductor	Annealing Temperature (°C)	Maximum Hole Mobilities (cm^2^/(V s))	Average Hole Mobilities ^1^ (cm^2^/(V s))	V_th_ (V)	I_on/Ioff_
P2TVDPP	150	0.41	0.38	3.40	9.71 × 10^6^

^1^ Measurement results based on eleven OFET devices.

## Data Availability

Data are included within the article and Supplementary Materials.

## Supplementary Materials

The following supporting information can be downloaded at: https://www.mdpi.com/article/10.3390/polym15234546/s1, Figure S1: ^1^H NMR spectra of compound **2**; Figure S2: ^13^C NMR spectra of compound **2**; Figure S3: ^1^H NMR spectra of compound **3**; Figure S4: ^13^C NMR spectra of compound **3**; Figure S5: ^1^H NMR spectra of compound **4**; Figure S6: ^13^C NMR spectra of compound **4**; Figure S7. ^1^H NMR spectra of monomer TVDPP; Figure S8: ^13^C NMR spectra of monomer TVDPP; Figure S9: GPC characterization for P2TVDPP.
