# Supplementary material for: Novel Divinyl-Flanked Diketopyrrolopyrrole Polymer, Based on a Dimerization Strategy for High-Performance Organic Field-Effect Transistors
Source: Polymers (Basel). 2023 Nov 27;15(23):4546. doi: 10.3390/polym15234546 (PMC10707771; doi:10.3390/polym15234546)
Supplement: Supplementary file 1 [file polymers-15-04546-s001.zip › polymers-2728432-supplementary.pdf]

Supplementary Information

Figure S1. ^{1}H NMR spectra of compound 2

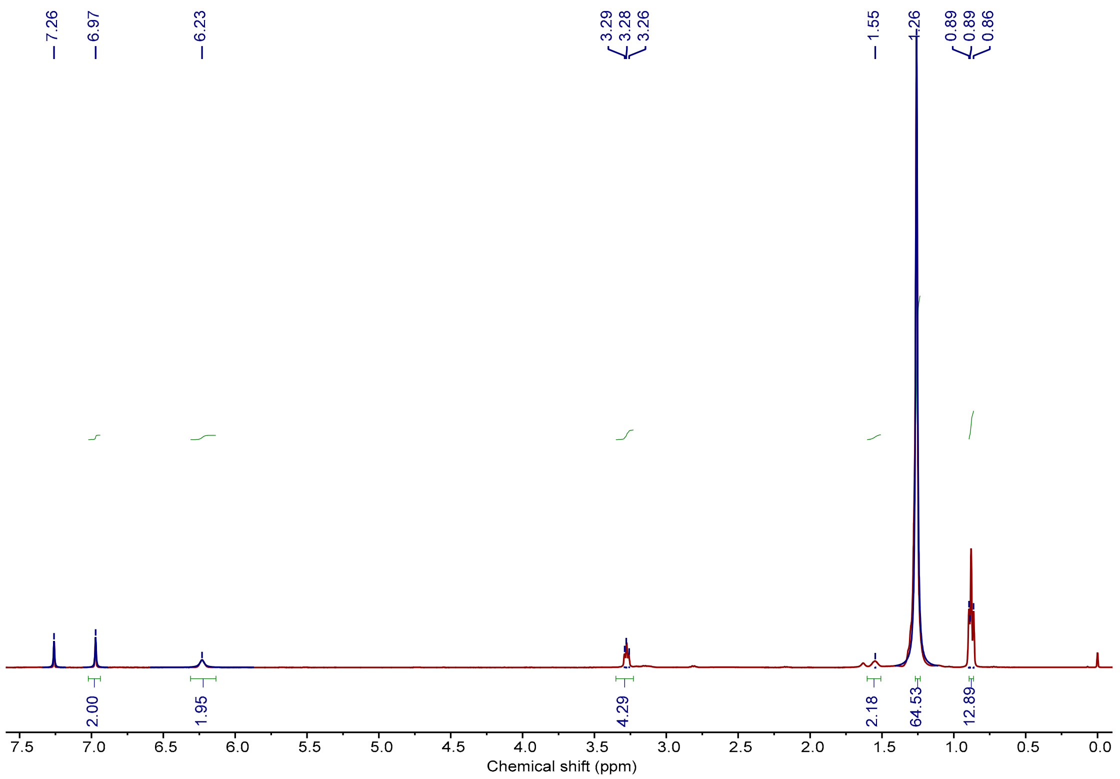

Figure S2. ^{13}C NMR spectra of compound 2

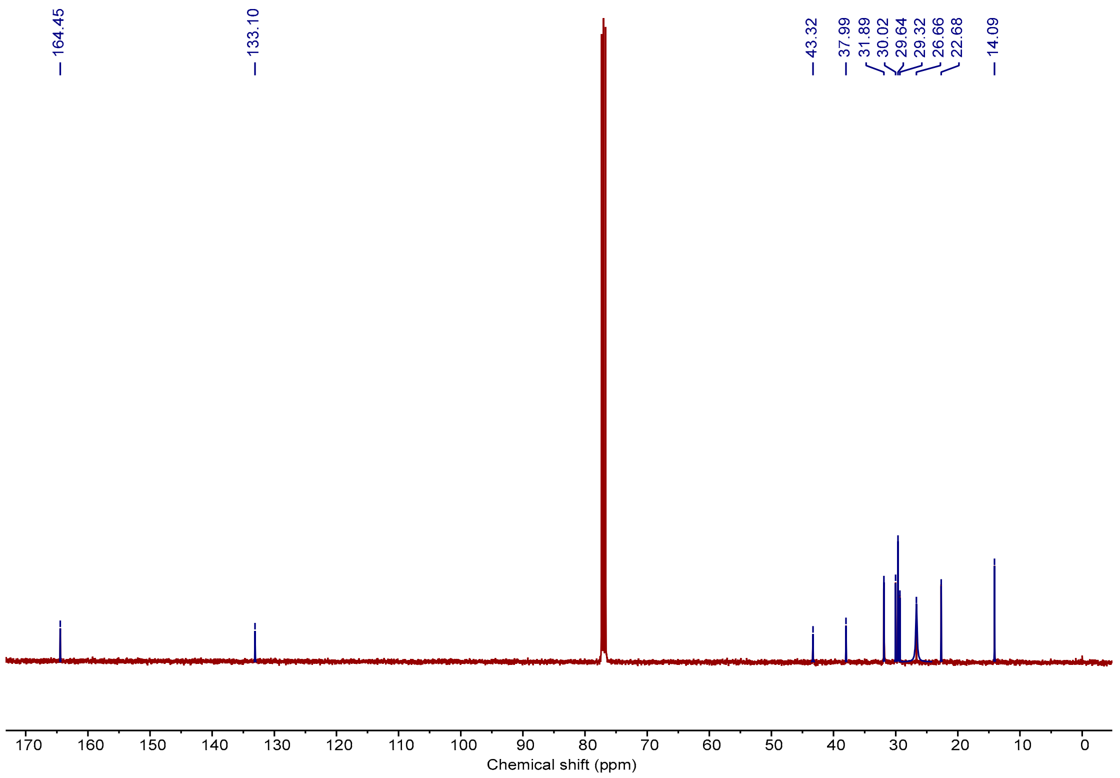

Figure S3. ^{1}H NMR spectra of compound 3

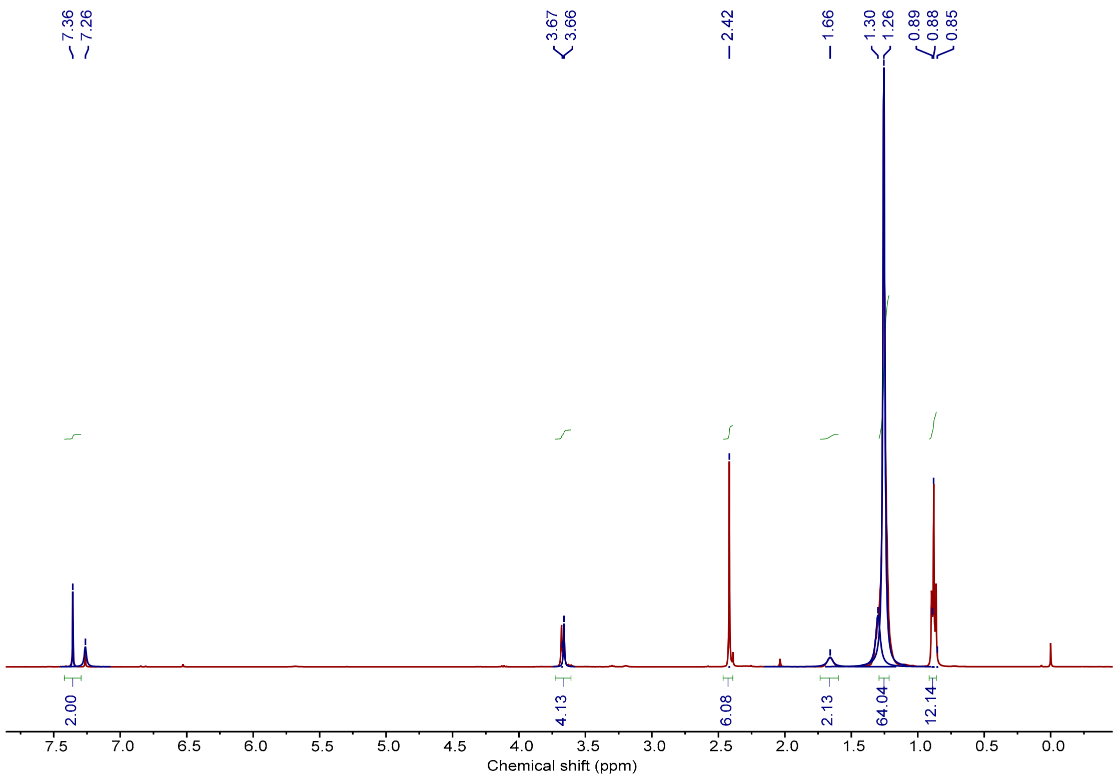

Figure S4. ^{13}C NMR spectra of compound 3

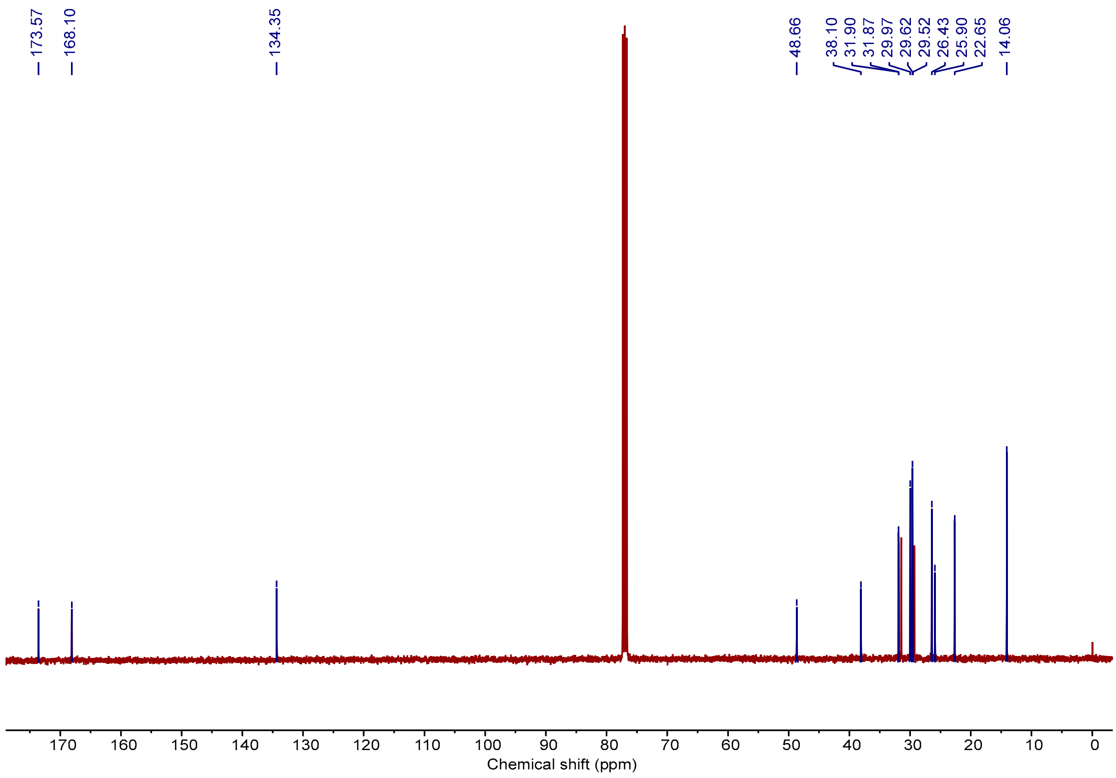

Figure S5. ^{1}H NMR spectra of compound 4

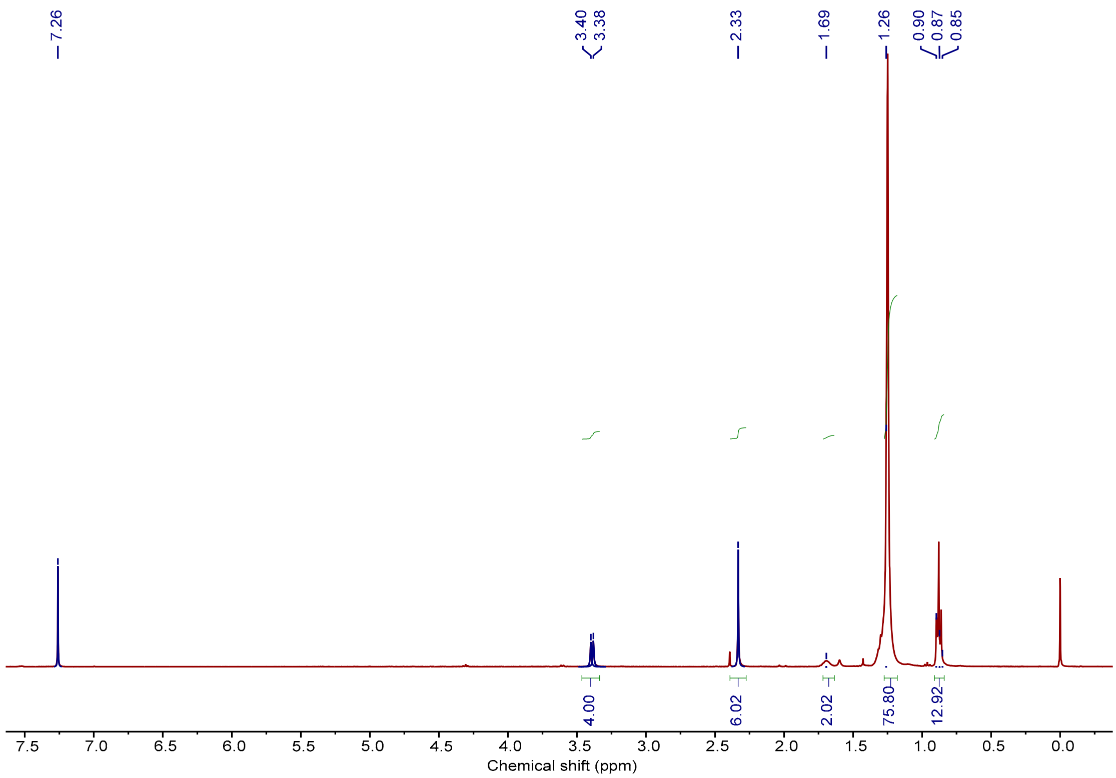

Figure S6. ^{13}C NMR spectra of compound 4

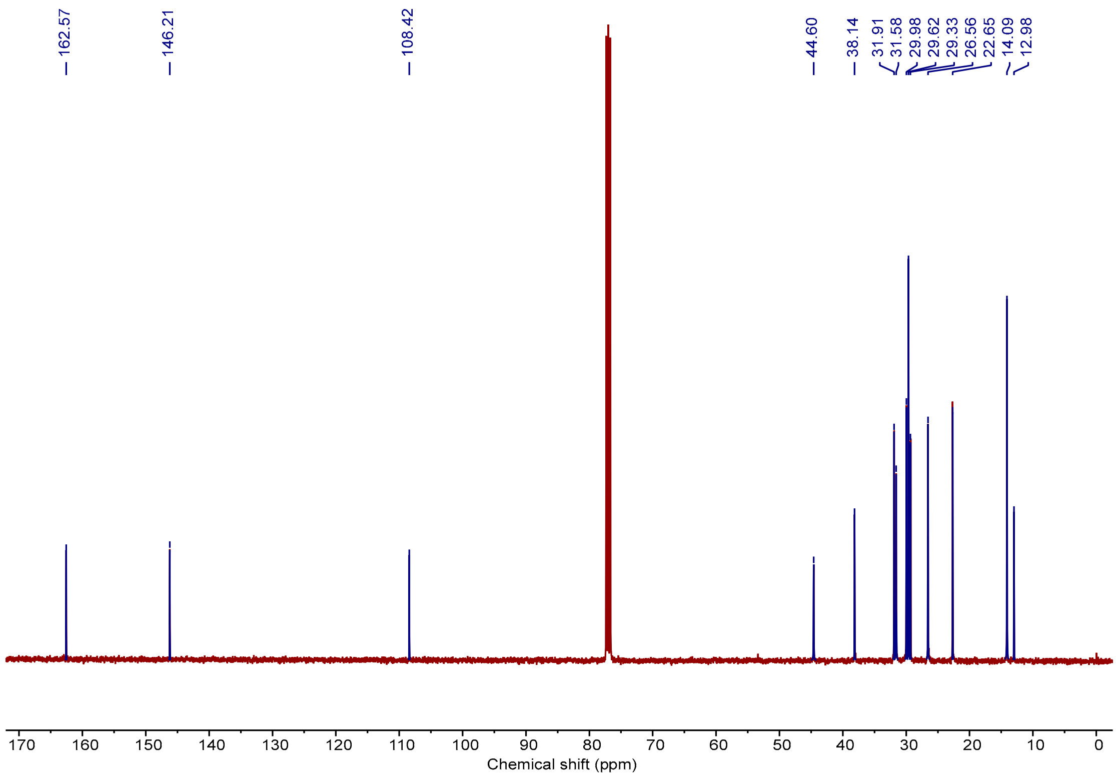

Figure S7. ^{1}H NMR spectra of monomer TVDPP

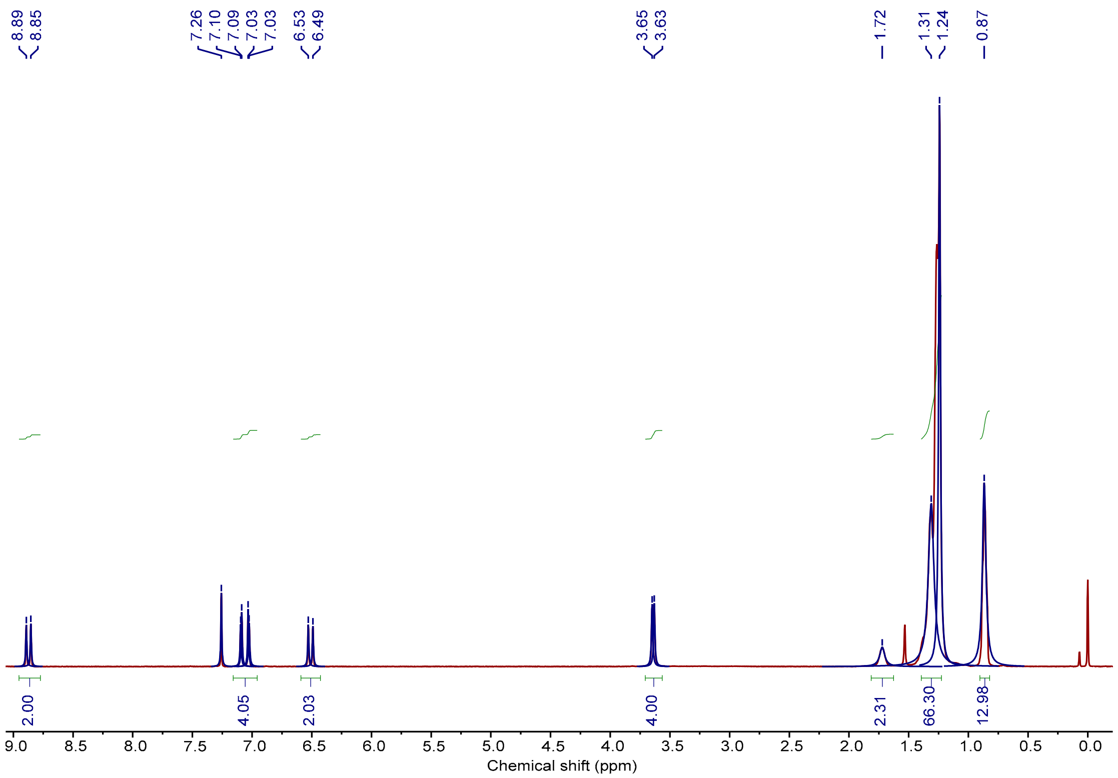

Figure S8. ^{13}C NMR spectra of monomer TVDPP

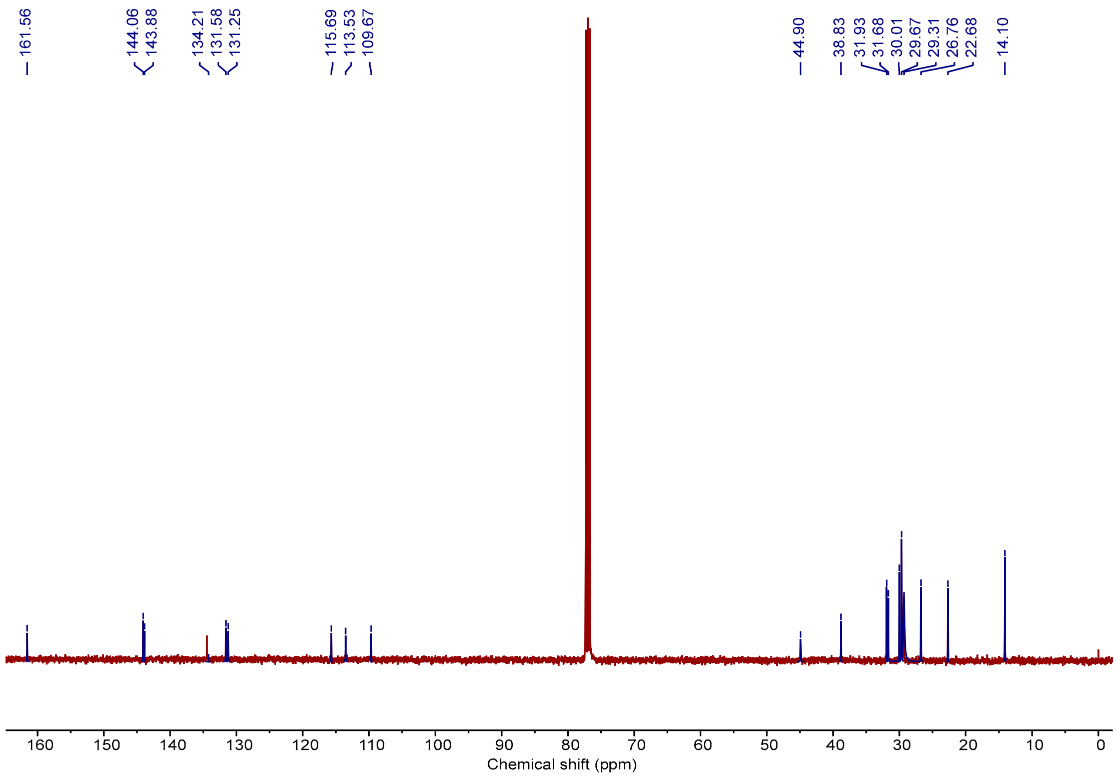

Figure S9. GPC characterization for P2TVDPP.

MW Averages

Mp: 59225	Mn: 35579	Mv: 61965	Mw: 67094
Mz: 105389	Mz+1: 142671	PD: 1.8858	

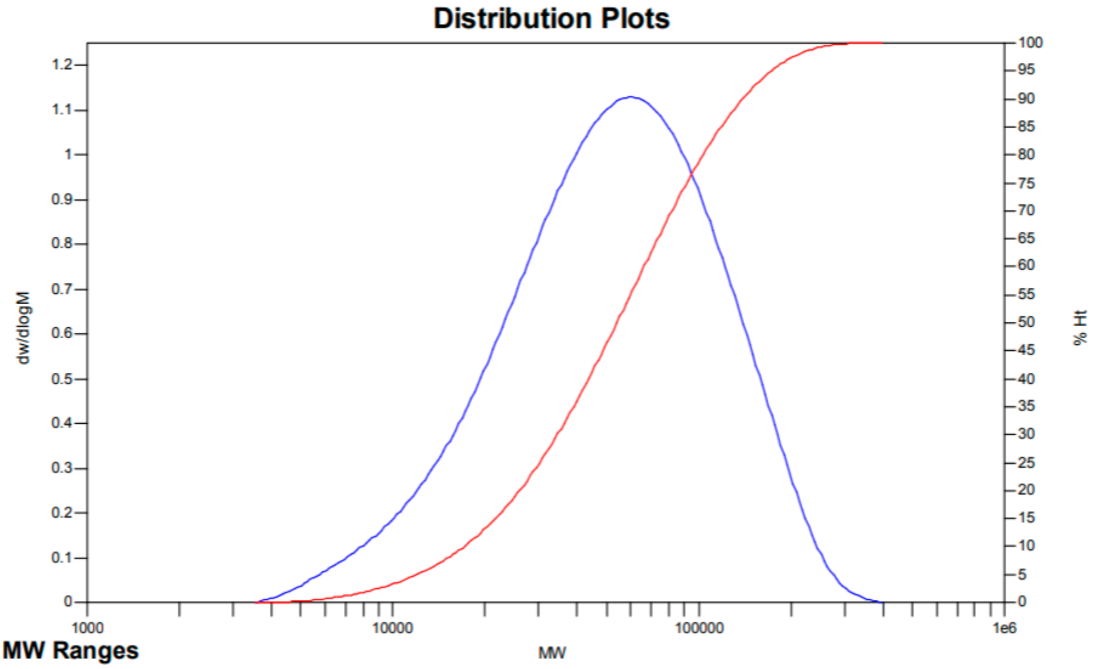

MW Ranges
